# Finite element analysis of a novel anatomical plate in posterolateral plateau fractures

**DOI:** 10.3389/fsurg.2024.1346462

**Published:** 2024-07-15

**Authors:** Zhen Jian, Xinhua Jiang, Dejian Li, Jianhua Zhou, Baoqing Yu, Chengqing Yi

**Affiliations:** ^1^ Department of Orthopedics, Shanghai Pudong Hospital, Fudan University Pudong Medical Center, Shanghai, China; ^2^ Department of Orthopedics, Seventh People's Hospital of Shanghai, Shanghai, China

**Keywords:** finite element analysis, tibial plateau fracture, plate, screw, biomechanic study

## Abstract

**Objective:**

This study aims to analyze the biomechanical characteristics of posterolateral plateau fractures fixed by a novel anatomical plate using finite element analysis.

**Methods:**

A three-dimensional digital model of the full length of right tibiofibula was obtained by CT scanning. A posterolateral tibial plateau fracture model was then created. The acquired fracture model was assembled with 4 groups of internal fixations: Group A, novel anatomical plate; Group B, straight buttress plate; Group C, oblique T-shaped locking plate; Group D, two lag screws. Axial loads of 500, 1,000 and 1,500 N perpendicular to the horizontal plane were used to simulate the stress on the lateral plateau of a 65 kg person standing, walking and fast running.

**Results:**

Vertical displacements of the posterolateral fragments in each of the four groups gradually increased under loads from 500 N to 1,500 N. The maximum displacement of the fracture fragment in four groups were all located on the lateral side of the proximal part, and the displacement gradually decreased from the proximal part to the distal end. The maximum displacement values under the axial load of 1,500 N was in the following order: novel anatomical plate (1.2365 mm) < oblique T-shaped locking plate (1.314 mm) < two lag screws (1.3747 mm) < straight buttress plate (1.3932 mm). As the axial load increased, the stress value of the different internal fixation models gradually increased. The stress behavior of the same internal fixation model under different loads was similar. The maximum stress value under the axial load of 1,500 N was in the following order: novel anatomical plate (114.63 MPa) < oblique T-shaped locking plate (277.17 MPa) < two lag screws (236.75 MPa) < straight buttress plate (136.2 MPa).

**Conclusion:**

The patients with posterolateral plateau fractures fixed with a novel anatomical plate in standing, walking and fast running can achieve satisfactory biomechanical results, which lays the foundation for future applications. At the same time, clinical fracture types are often diverse and accompanied by damage to the soft tissue. Therefore, the ideal surgical approach and appropriate internal fixation must be selected based on the patient's injury condition.

## Background

Posterolateral plateau fractures account for up to 11.7%–15.6% of all tibial plateau fractures (1, 2). It is usually difficult to be detected based on x-ray images of the knee joint alone. With the widespread use of computed tomography (CT), it has been reported that, 54.3% of lateral plateau fractures involve the posterolateral plateau (3), and 44.32% of bicondylar fractures involve the posterolateral plateau (4). Posterolateral tibial plateau fractures are also intra-articular fractures that generally require open reduction and internal fixation. The purpose of operation is to restore the alignment of the lower limb and restore the flatness of the joint surface.

The exposure is often blocked by the fibular head, which increases the difficulty of treatment. For slightly displaced fractures, two lag screws from anterior to posterior are usually used for the fixation. For the fractures requiring reduction, the posterolateral approach without fibular osteotomy can well expose the posterolateral fracture line in most cases without disturbing the stability of the knee joint and the common peroneal which is reported to achieves good reduction and clinical results (5). A posterolateral buttress plate or oblique T-shaped plate is commonly inserted through the approach. Based on the anatomical shape of the posterolateral tibial plateau, a novel anatomical plate was designed to fix the fracture through posterolateral approach, and previous clinical study has proven that the plate is safe and effective in 12 patients during a 12–34-month follow-up (6). However, there is still a lack of comparison of the effects of the novel fixation method with currently common-used fixations. The effect of surgical fixation is closely related to the biomechanical properties of the internal fixation. Finite element analysis is one of the most powerful mechanical property analysis tools in biomechanical research (7). It can simulate geometric models of various structures, assign biomaterial properties to various tissues, and can well reflect the overall trend of their biomechanical properties.

Therefore, the purpose of this study is to compare the biomechanical stability of the novel posterolateral anatomical plate of tibial plateau with three other commonly used clinical internal fixations through the finite element method, thereby providing a theoretical basis for clinically selecting appropriate internal fixation to treat the posterolateral tibial plateau.

## Methods

The CT scan data conducted through 0.625 mm slice thickness for the full length of right tibiofibula on a healthy adult male (27 years old, 175 cm in height, 65 kg body weight) without comorbidities, such as instability, fracture, degeneration, and osteoarthritis, was obtained and used to construct a three-dimensional tibiofibula finite element model using Mimics software (v21.0, Materialize Company, Belgium). the initial model was further improved using Geomagic Studio Software (v2017, 3D system Inc., Rock Hill, USA).

**Figure 1:**
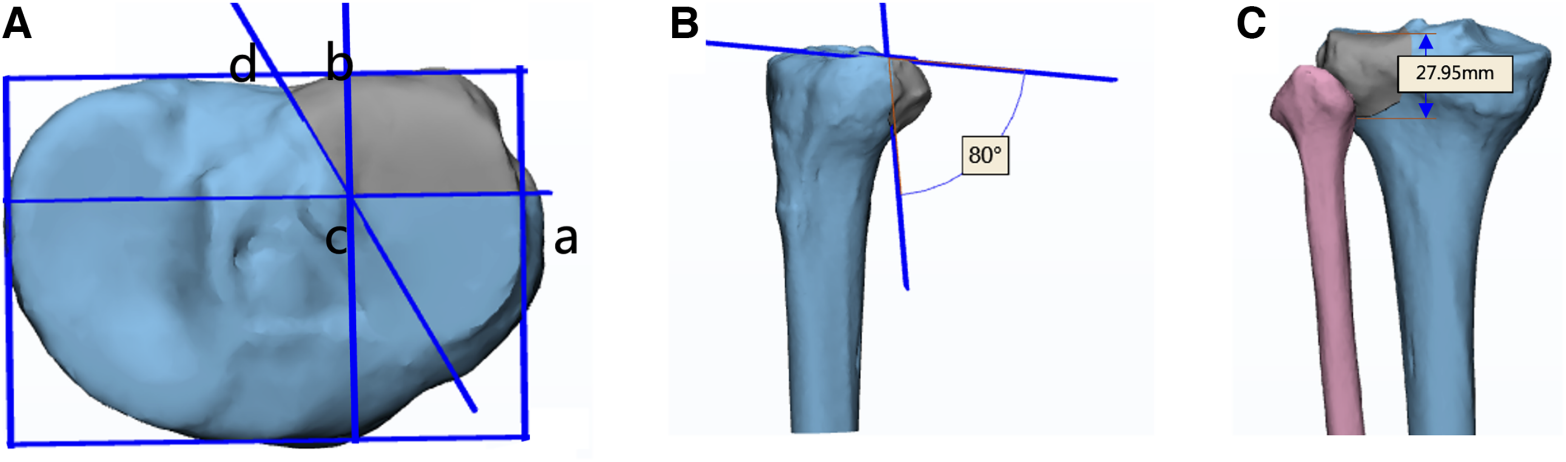
Model of posterolateral column fracture of the tibial plateau. (**A**) the angle between fracture line ac and cd on the cross plane is 120°; (**B**) the angle between the fracture line and the articular surface in the sagittal plane is 80°; (**C**) the distance from the articular surface to the distal point of the fracture line is 27.9 mm.

In addition, a posterolateral tibial plateau fracture model was created as previously reported (8) using SolidWorks (v2017, Dassault Systemes, S.A, Paris, France). The detailed method was as follows: A rectangle was made based on the maximum left-right diameter of the tibial plateau and the maximum anteroposterior diameter of the lateral plateau. The intersection of the posterior 1/3 of the tibial plateau and the lateral line of the rectangle was at Point a, and the intersection of the lateral 1/3 of the tibial plateau and the posterior line of the rectangle was at Point b. The perpendicular lines passing through a and b intersected at Point c. Then a line was made through Point c which angles at 120° with the straight-line ac. Since the tibial plateau has a posterior slope angle of about 10°, sagittal angle between posterolateral fracture fragment and plane of articular surface of tibial plateau was set 80°. On the coronal plane, the distance from the fracture line of the articular surface to the most distal end was 27.95 mm, which was very close to the average value of 28 mm reported in the previous study (9). (Shown in Figure 1).

**Figure 2:**
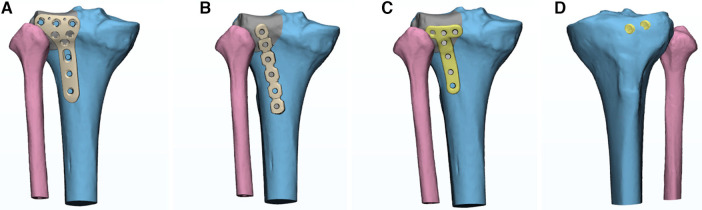
Internal fixation models of four groups. (**A**) Group A: novel anatomical plate group; (**B**) Group B: straight buttress plate group; (**C**) Group C: oblique T-shaped locking plate group; (**D**) Group D: two lag screws group.

The acquired posterolateral fracture model of the tibial plateau was assembled with 4 groups of internal fixations on the assembly interface using the SolidWorks. The internal fixation was fixed at 5 mm below the tibial plateau using the isometric outer surface of the tibia (10). The screws were set as smooth cylinders, and 4 groups of internal fixation models were obtained, as shown in Figure 2.

The assembled 4 groups of internal fixation models were imported into Ansys (v17.0, ANSYS, Inc., Canonsburg, PA, USA) for analysis. The tibia, plates and screws were set as homogeneous and isotropic linear elastic materials in the study. In terms of boundary conditions, the distal tibia was set as the translation and rotation of the distal tibial underside was constrained in three dimensions. The models of the volume meshes were imported into Mimics software to endow material properties. The material properties of tibia, plates and screws were set according to relevant references (7, 11), as shown in Table 1. The friction coefficient of the fracture surface was set to 0.4 (12). Plates and screws were constructed as rigid connections through common nodes of shared elements. The grids of the 4 groups of internal fixation models were optimized and set, and the numbers of elements and nodes of various models in the experiment were shown in Table 2.

**Table 1:** Material properties of each part of the skeleton model and internal fixations.

Area and material	Young's modulus (MPa)	Poisson's ratio
Cortical	18,000	0.3
Cancellous	500	0.2
Internal fixations	110,000	0.3

**Table 2:** Numbers of fixation model nodes and elements of each group.

Model	Nodes	Elements
Group A	368,848	215,618
Group B	217,480	120,895
Group C	313,345	181,115
Group D	160,403	85,688

Previous studies have shown that under normal gait, the biomechanical load on the knee joint is approximately two to three times of the body weight (13, 14). When walking, mechanical axis of the lower limb is biased towards the medial condyle, so the medial plateau of the tibia has a larger stress area accounting for 60%, and the lateral plateau accounts for 40% of the stress. In our study, axial loads of 500, 1,000 and 1,500 N perpendicular to the horizontal plane were used to simulate the stress on the lateral plateau of a 65 kg person standing, walking and fast running (15).

Main Outcome Indicators included displacement distribution and maximum displacement of model fracture fragments in each group, as well as stress distribution and peak value of internal fixation.

## Results

**Figure 3:**
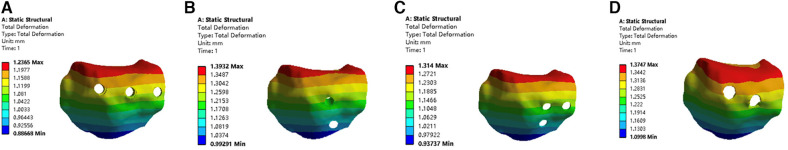
Vertical displacement of the posterolateral fragments of the four groups under an axial load of 1,500 N. (**A**) novel anatomical plate group; (**B**) straight buttress plate group; (**C**) oblique T-shaped locking plate group; (**D**) two lag screws group.

Vertical displacements of the posterolateral fragments in each of the four groups gradually increased under loads from 500 N to 1,500 N (Table 3). As the axial load increased, the displacement nephograms of the fracture fragments in each group showed similar distribution characteristics. Therefore, the article only provided the fracture fragment displacement cloud diagram under the axial load of 1,500 N, as shown in Figure 3. The maximum displacement of the fracture fragment in four groups were all located on the lateral side of the proximal part, and the displacement gradually decreased from the proximal part to the distal end. Under the load of 500–750–1500 N, the fracture fragment displacement of the novel anatomical plate group was the smallest, while the fracture fragment displacement of the straight buttress plate group was the largest. The maximum displacement values under the axial load of 1,500 N were in the following order: novel anatomical plate group (1.2365 mm) < oblique T-shaped locking plate group (1.314 mm) < two lag screws group (1.3747 mm) < straight buttress plate group (1.3932 mm).

**Table 3:** Maximum displacement of posterolateral fragments under different loads of each group.

Model	Maximum displacement (mm)		
	500 N	1,000 N	1,500 N
Group A	0.41	0.82	1.23
Group B	0.46	0.93	1.39
Group C	0.44	0.88	1.31
Group D	0.46	0.92	1.37

**Figure 4:**
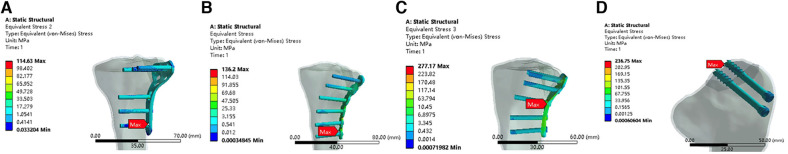
Stress distribution of the four groups under an axial load of 1,500 N.(**A**) novel anatomical plate group; (**B**) straight buttress plate group; (**C**) oblique T-shaped locking plate group; (**D**) two lag screws group.

As the axial load increased, the stress value of the different internal fixation models gradually increased (Table 4). Since the stress behavior of the same internal fixation model under different loads was similar, the article only provided the stress distribution cloud diagram of the internal fixations under the load of 1,500 N, as shown in Figure 4. The maximum stress of the fixation in the novel anatomical plate group was around the most distal screw hole, and the stress distribution is relatively uniform. The stress of the straight buttress plate group was mainly focused on the tails of four distal screws and the local area of the plate between them, and the maximum value was around the most distal screw hole. The stress distribution in oblique T-shaped locking plate group was focused on local areas of the connection between the plate head and the stem and on the screw located while the maximum stress was found on the middle screw hole. In the two lag screws group, the stress maximum was mainly focused on head closing to the end cap. The maximum stress value under the axial load of 1,500 N were in the following order: novel anatomical plate group (114.63 MPa) < oblique T-shaped locking plate group (277.17 MPa) < two lag screws group (236.75 MPa) < straight buttress plate group (136.2 MPa).

**Table 4:** Maximum stress experienced by internal fixation devices under different loads of each group.

Model	Maximum stress (MPa)		
	500 N	1,000 N	1,500 N
Group A	38.21	76.42	114.63
Group B	45.01	90.80	136.2
Group C	92.39	184.78	277.17
Group D	78.91	157.83	236.75

## Discussion

The prognosis of tibial plateau fractures is related to the quality of anatomical reduction of the joint surface and stable osteosynthesis to enable early knee mobilization (5). Currently, there are two difficulties in treating posterolateral tibial plateau fractures. One is the surgical approach. The posterolateral tibial plateau is surrounded by the fibular head, posterolateral complex (PLC), common peroneal nerve, popliteal nerve and vessels. Access to the posterior region of the knee is often considered a difficult task due to the depth of the operative field and the presence of vascular and nervous elements that pass through it. It is hard to achieve effective reduction through the anterolateral surgical approach which is most familiar to orthopedic surgeons. Approaches used in posterior tibial plateau fractures (PTPFs) have undergone significant changes in recent years (16–18). However, if the posterolateral surgical approach is chosen, the surgeons will face the second difficulty, that is the choice of internal fixation method. The existing internal fixation methods cannot achieve sufficient and effective fixation, and inappropriate internal fixation will inevitably increase the detachment of the posterolateral knee joint structure, increasing the risk of blood vessel and nerve damage.

The anterolateral approach is the most commonly used and familiar surgical approach by orthopedic surgeons because it is safe and simple to operate. After fracture reduction, two lag screws from anterior to posterior are usually used fixation method. The fixation can be completed through the anterolateral approach and is mostly used for large, complete posterolateral fractures. However, it is difficult to directly expose the posterolateral fracture through this approach. Although the posterolateral articular surface can be directly viewed through the joint space above the fibular head, it is difficult to maintain reduction. When the posterolateral fracture is comminuted or the bone fragment is small, sometimes only one screw can be fixed to the posterolateral fracture fragment, and it is rather difficult to fix the fracture effectively. Previous research found that compared with lateral anatomic 3.5 mm L-shaped locking plate, two lag screws and posterolateral 3.5 mm six-hole straight buttress plate fixations of posterolateral tibial plateau fractures achieved less stability (10). This was similar to the results of the current study.

The posterolateral approach can directly expose the posterolateral plateau area. During the operation, the posterior split bone fragments are lifted up. After the collapsed tibial platform is raised and reduced, autogenous bone or artificial bone can be filled in. The approach facilitates reduction and bone grafting which also helps the maintenance of reduction. During the operation, the lateral head of the gastrocnemius muscle is stretched medially, and the biceps femoris muscle and common peroneal nerve are stretched lateral to protect the PLC of the knee joint. Huo et-al (19) followed up 200 patients with posterolateral fractures of the tibial plateau for 1 year after surgery, and compared the advantages and disadvantages of the posterolateral approach with the anterolateral approach, and concluded that it is more suitable for some comminuted posterolateral fractures. He reported the posterolateral approach increased the HSS score by 6.1 points compared with the anterolateral approach when the diameter of the fracture fragment was less than 20 mm.

After reduction, a straight buttress plate or an oblique T-shaped locking plate is usually used for support and fixation. The posterolateral buttress plate has been proven to achieve “adequate stability” for posterolateral shearing tibial plateau fracture as the vertical displacement of the fragment under axial load was no more than 1 mm (8). However, the fixation methods are not anatomical plates which require reshaping to ensure that the plates are fit to the posterior surface of the tibia, and special attention must be paid to avoid damage to the anterior tibial artery. The study showed the stress distribution of novel anatomical plate was relatively uniform. Based on previous studies (20, 21), a displacement of 2 mm was regarded as acceptable and a displacement that could shorten the healing time with a small range of motion. Naturally, the smaller the maximum displacement, the greater the postoperative stability of the fracture. As a result, the novel anatomical plate provides adequate mechanical stability, and the overall stability is higher than the other three sorts of fixations.

The novel anatomical plate better fits the shape of the posterolateral tibial plateau. The proximal end of the plate wraps the posterolateral plateau without being restricted by the fibular head. The proximal universal screw-hole design can ensure that the screws are implanted in parallel to the joint surface, thereby achieving the “raft effect” to effectively support the articular surface. This finite element analysis showed the plate achieved better biomechanical properties. The short design of the modified plate can avoid injury to the branches of the posterior tibial artery. MAY (22) measured the distance between the point where the anterior tibial artery passes through the interosseous membrane and the tibial plateau and the top of the fibula on digital subtraction angiography images of 219 lower limbs, which were (50.9 ± 6.9) and (36.5 ± 6.0) mm, respectively. Moreover, the anterior tibial artery has poor mobility due to the traction of the interosseous membrane fibers, so the length of the plate should be especially paid attention for the posterolateral approach.

The limitations of this study are as follows. The experiment gave a single axial static load, but the real tibial plateau force is multi-directional and dynamic (23), and the failure of fracture fixation may also be caused by repeated application of load. The finite element model is an ideal model and ignores the impact of soft tissue on the surrounding Impact, only consider the excellence of the internal fixation from biomechanics. The fracture model is established as an ideal single split fracture, but clinically there are various types of fractures, and they are often accompanied by damage to the soft tissue around the knee joint. Therefore, it needs to be based on select the ideal surgical approach and appropriate internal fixation according to the patient's injury condition. The experiment adopts the finite element analysis method, while ignoring the influence of the fibular support and failing to fully simulate the stress on the tibial plateau. Therefore, the next step will be to conduct biomechanical experiments on fake bone models and cadaver bone models to verify the finite element results.

## Conclusions

The results of the finite element analysis show that the novel anatomical plate has better biomechanical stability than the traditional commonly used straight buttress plate, oblique T-shaped locking plate and two lag screws when simulating the stress on the lateral plateau of a 65 kg person standing, walking and fast running. It shows good biomechanical stability, laying the foundation for its future applications. However, the clinical fracture types are often diverse and accompanied by damage to the soft tissue around the knee joint. Therefore, the ideal surgical approach and appropriate internal fixation must be selected based on the patient’s injury condition.

## Data Availability

The original contributions presented in the study are included in the article/Supplementary Material, further inquiries can be directed to the corresponding authors.
